# The effectiveness of percutaneous endoscopic lumbar discectomy combined with external lumbar drainage in the treatment of intervertebral infections

**DOI:** 10.3389/fsurg.2022.975681

**Published:** 2022-08-09

**Authors:** Qun Huang, Qi Gu, Jincheng Song, Fei Yan, XiaoLong Lin

**Affiliations:** Department of Orthopaedic Surgery, The Affiliated Zhangjiagang Hospital of Soochow University, Suzhou, China

**Keywords:** drainage, lumbar intervertebral infection, lesion clearance, percutaneous endoscopic lumbar discectomy, minimally invasive

## Abstract

**Objective:**

To analyze the effect of percutaneous endoscopic lumbar discectomy in treating lumbar intervertebral infections.

**Methods:**

A total of 13 patients with lumbar intervertebral infections who underwent percutaneous endoscopic lumbar discectomy combined with external drainage between November 2016 and December 2019 were enrolled in the present study. After the operation, sensitive antibiotics were used based on the results of the bacterial culture. If no pathogens were detected in the biopsy culture of the infected tissues, empirical antibiotics were administrated to these patients. The clinical efficacy was evaluated by using a visual analog scale (VAS), Japanese Orthopaedic Association (JOA), Oswestry Disability Index (ODI), and standard Macnab's evaluation. Postoperative computed tomography (CT) and MRI were also used to evaluate clinical efficacy.

**Results:**

The follow-up time was 10–18 months, and the average time was (13.69 ± 2.63) months. Causative bacteria were isolated in 7 of 13 infected tissue biopsy cultures. Systemic antibiotics and anti-tuberculous chemotherapy were administered according to sensitivity studies for identified. There were no pathogens isolated from the other six patients. Empiric antibiotics were administrated in these patients. One week after the operation, WBC, a fractional fraction of medium granulocytes, ESR and CRP were significantly lower compared to before the operation (all *P* < 0.05). At the last follow-up visit, the above-mentioned markers were all within normal range, which differed compared to the pre-operative data (*P* < 0.05). The VAS and ODI of the patients at 1 week and 3 months after operation were significantly lower compared to preoperative data (all *P* < 0.05). During the last follow-up visit, seven patients were excellent, five were good, and one was poor according to standard Macnab's evaluation. No serious complications were recorded.

**Conclusions:**

Percutaneous lumbar discectomy combined with external drainage resulted as an effective method for treating lumbar intervertebral infections and was associated with fewer injuries, less pain, low cost, and low recurrence rate.

## Introduction

Intervertebral disc infections include the infection of the intervertebral adjacent vertebral body and soft tissue (1–4). Low immunity, diabetes mellitus, tumors, and AIDS are the predisposing factors for intervertebral infection (5, 6). In addition, the number of iatrogenic infections after spinal injections or spinal surgery has been steadily increasing (7–9). The most common symptoms are intractable back pain, passive posture, and in some cases, nerve injury (10). Diagnosis for intervertebral space infection is based on clinical symptoms, laboratory studies such as elevated erythrocyte sedimentation rate (ESR), C-reactive protein (CRP) values and blood cultures, and roentgenographic and magnetic resonance image (MRI) findings with confirmation provided by histopathologic examination (11–13). ESR and CRP were shown to be significantly reliable in the identification of pyogenic spondylitis (11). Blood cultures are usually performed in patients with infectious spondylitis to identify the infecting agent, but the detection rate is only 18% to 58% (13). In addition, most MRI criteria commonly used to diagnose disk infections offer good to excellent sensitivity. In atypical manifestations of proven spinal infections, however, some of the classically described MR imaging criteria may not be observed (12). Furthermore, computed tomography (CT)-guided needle aspiration biopsy was the most common procedure for bacteriologic diagnosis. However, needle biopsy for bacteriological diagnosis has been reported to have a variable success rate, ranging from 38% to 73% (14). In fact, the most reliable tests for finding the causative infectious agents are histological examinations and cultures of the samples taken from the infection sites. Although bacterial culture is the gold standard for the diagnosis of infectious spondylitis, it is not necessary for early diagnosis and prompt treatment. Once lumbar infectious spondylitis is suspected, broad-spectrum antibiotics should be initiated immediately (15). The clinical manifestations may vary with different sites of infection, a number of affected segments and pathogenic bacteria, as the anatomical location of intervertebral disc infection is deep and blood culture is not sensitive. Moreover, the particularity of the anatomical structure of the vertebral intervertebral space makes it difficult to achieve an effective concentration of antibiotics. Consequently, treating such patients is difficult, time-consuming and expensive.

With the increase of lumbar surgery cases, intervertebral disc infections have also been increasing, thus seriously affecting the surgical outcome and the recovery process of patients (16). For most patients, conservative treatment can significantly relieve all of the symptoms (17, 18). However, surgical treatment is required for patients who fail to respond to conservative treatment and present with symptoms of nerve compression, large abscesses or paraspinal abscess formation, infection involving adjacent vertebral bodies, and progressive deformities of the lumbar spine (19, 20). The purpose of surgical treatment is to completely debride, relieve nerve compression, identify pathogenic bacteria and restore the stability of the spine (21, 22). The main surgical treatment methods include traditional incision, debridement, washing and drainage, internal fixation, and CT-guided puncture and drainage (18, 19). Traditional surgery can be performed by using the posterior approach, anterior approach or combination of the two, which are used to remove the lesions and intervertebral fusion, thus achieving positive results (23, 24). However, the operation is usually lengthy, the trauma is substantial and the postoperative recovery is slow.

Some patients cannot tolerate incision and drainage due to poor health conditions and a high risk of anesthesia. Over recent years, with the development of minimally invasive percutaneous endoscopic techniques, percutaneous endoscopic discectomy (PED), a simple, safe, and minimally invasive approach, has been increasingly accepted by a number of researchers. Some studies also reported that PED could be applied in managing spinal infections (25, 26). Herein, we reviewed 13 cases of patients with lumbar intervertebral infections treated by PED combined with external drainage in our hospital between November 2016 and December 2019 and discussed the efficacy of PED in the treatment of lumbar intervertebral infections.

## Material and methods

### Clinical data

The study design was a retrospective cases series and it was approved by the medical ethics committee of the authors' institution. All patients signed written informed consent when they entered the study. The image data and intraoperative images included in the paper were approved by the patients. Also, the informed consent signed by the patients indicates that they can also be used in subsequent clinical studies.

A total of 13 patients with lumbar intervertebral infections, 5 males and 8 females, aged from 45 to 69 years (average of 58.27 ± 7.83 years), were enrolled in the present study between November 2016 and December 2019. Lumbar intervertebral infection was diagnosed based on clinical examinations, including elevated erythrocyte sedimentation rate (ESR) and C-reactive protein (CRP) values and radiographic and MRI findings. In addition to these tests, all patients underwent blood culture and puncture biopsy prior to surgery. All cases were single-level lumbar intervertebral infections. They also had a variety of comorbidities, including renal failure, heart failure, rheumatic arthritis and diabetes (Table 1). Inclusion criteria were the following: (A) intractable back pain, which could not be relieved by conservative therapy; (B) MRI showed abscess formation in the intervertebral space or spinal canal; (C) the lesion was confined to a single space or a single vertebral body; (D) imaging showed obvious dural sac or neurological deficit. Exclusion criteria were: (A) spinal deformity and instability; (B) severe destruction of vertebral bone; (C) MRI indicated that the abscess was located in the spinal canal subdural; (D) epidural abscesses larger than 2 levels or 2 intervertebral spaces; (E) patients with obvious abnormal coagulation function or lack of coagulation factors.

**Table 1:** Information of patients.

Patient no.	Gender	Age	Level	Therapy method	Follow-up (months)	Complication	Associated medical illness
1	Male	51	L4-5	PLD	13	None	None
2	Female	58	L5-S1	PLD	16	None	HTN, RA
3	Female	45	L4-5	PLD	17	None	HTN, CHF
4	Male	61	L4-5	PLD	14	Paresthesia	DM, HTN
5	Male	69	L2-3	PLD	18	None	RHD, asthma
6	Female	67	L4-5	PLD	16	None	DM
7	Male	53	L3-4	PLD	12	None	HTN, CHF
8	Male	63	L4-5	PLD	10	None	TB, CAD
9	Female	65	L4-5	PLD	12	None	DM
10	Female	50	L5-S1	PLD	11	None	DM
11	Female	68	L3-4	PLD	15	None	CAD, HTN, DM
12	Female	58	L3-4	PLD	14	None	HTN, DM
13	Female	50	L4-5	PLD	10	None	None

CHF, congestive heart failure; RHD, rheumatic heart disease; CAD, coronary artery disease; DM, diabetes mellitus; HTN, hypertension; RA, rheumatoidarthritis; and TB, tuberculosis.

A total of 13 patients were treated under local anesthesia with PED clearance, irrigation, and drainage. The operation was performed by the same surgeon. The JOIMAX system was used (JOIMAX GMBH, Karlsruhe, Germany). According to the results of bacterial culture, sensitive antibiotics were given for anti-inflammatory treatment. Erythrocyte sedimentation rate and C-reactive protein were monitored to evaluate disease management.

### Surgical techniques

PED Technique, preoperative CT, MRI, and other related examinations were performed to identify the infection space and site. The patient was lying on the operating table on the side, and the level of the intervertebral foramen and intervertebral space was located by C-arm fluoroscopy. Under the guidance of fluoroscopy, the target site was located, and the approach site was marked 8 cm–12 cm from the midline of the skin. After aseptic preparation and local anesthesia (2% lidocaine), the spinal needle was inserted directly into the center of the target intervertebral disc. The guidewire was introduced through the spinal needle into the central intervertebral disc space, and the spinal needle was removed.

After creating a small puncture incision (about 1 cm), an expander and a hollow cannula were sequentially guided across the wire and into the center of the disc. The continuous expansion was achieved by casing (Jinan Longguan Company, China). The fluoroscopy was repeated in two orthogonal planes to confirm the correct position of the endoscope tip.

Next, the tissue expander was removed, and the cutting tool, i.e., a cylindrical sleeve with a distally serrated edge used to obtain the core of a biopsy specimen, was inserted. A nucleus pulposus bone-biting forceps (Chinese dragon crown) was then inserted into the disc through the cannula to extract the purulatory material and bone marrow tissue as slowly as possible. Under fluoroscopic monitoring and endoscopy, the greatest possible quantity of tissue was removed by placing biopsy forceps, flexible biting forceps, and a razor in different positions. During surgery, the infected disc was fully removed and extensively debrided, and even some endplates were removed from different endoscopic directions. The necrotic tissue and abscess wall around the lesion were removed with bipolar radiofrequency electrotome until the surrounding tissue with good blood flow appeared. A high-voltage bipolar probe was used for thermocoagulation of infected tissue and bleeders. After adequate debridement, at least 4L of normal saline solution was used to irrigate the surgical field until healthy bone of upper and lower vertebral bodies were visible. Finally, when there was no obvious compression of nerve root and dural sac, the procedure ended. The biopsy specimen included disc materials. The microbiological examination was performed on each biopsy specimen, followed by a histopathological evaluation. Postoperatively, normal saline was rinsed with an external drainage catheter.

**Figure 1:**
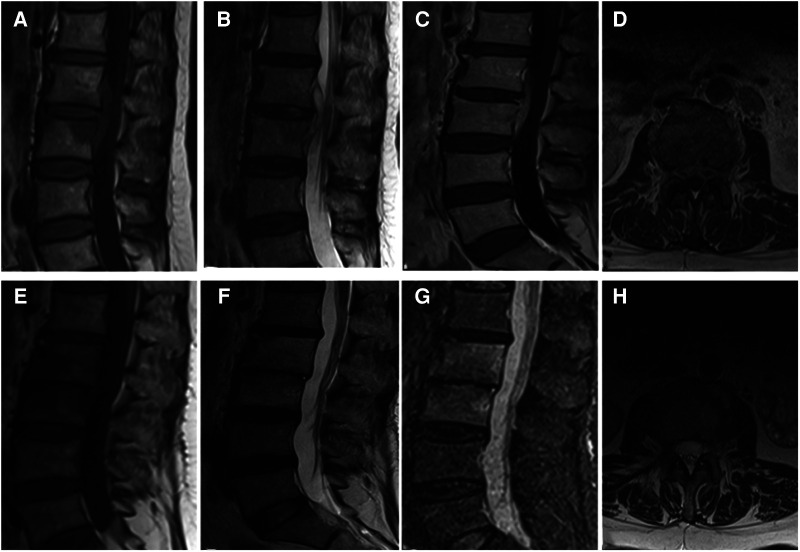
(**A–D**) It presents the MRI scans of patient No. 9. Sagittal and axial magnetic resonance images with L2-L3 intervertebral infection. (**E–H**) It presents the postoperative the MRI scans (6 months after surgery) of patient No. 9. MRI shows that the abscess was disappeared without recurrence. (**A,E**) Sagittal T1WI. (**B,F**) Sagittal T2WI. (**C,G**) Sagittal contrast-enhanced image. (**D,H**) Axial image.

Another external drainage catheter was attached to the drainage bag, and the drainage was maintained. Two drains were placed into the intervertebral disc space through the cannulated sleeve under fluoroscopic monitoring and endoscopic view. In brief, drainage tube was put through the skin next to the incision after flushing tube was inserted into the debrided disc space. It should be noted that the two drains could not pass through the skin too close together to avoid leakage. Inputs and outputs were recorded on a daily basis. Systematic antibiotic therapy was also given. The vertebral bodies were examined by CT (Toshiba Aquilion, Japan) and MRI (GE HDXT-3T, GE, United States). A representative case is shown in Figure 1.

### Postoperative treatment

Effective antibiotics were administered intravenously for patients with known causative pathogens before surgery. For patients with unknown pathogens, empirical antibiotics were administered immediately after surgery. These were switched to specific antibiotics after identification of the causative pathogen was made from intraoperative tissue or pus culture. Intravenous antibiotics were used for 4 to 6 weeks according to follow-up inflammatory markers. The patients were then switched to oral antibiotics and discontinued when the inflammatory markers were within a normal range. The patient remained in bed for 2 weeks after surgery. A rigid thoracolumbar spinal orthosis was then used for ambulation.

All patients were continuously irrigated with 0.9% normal saline containing gentamicin for 5 days(2000 ml/24 h). The criteria for stopping drainage tube flushing: (1) the drainage fluid became clear and the patient's symptoms disappeared; (2) erythrocyte sedimentation rate [ESR], C-reactive protein [CRP] were significantly decreased; (3) the bacterial culture of blood and flushing fluid is negative for 2 consecutive times. The drainage tubes were retained in place until the daily drainage had stopped or reduced to less than 5 ml per day for 3 consecutive days.

During bed rest, the patients were encouraged to perform early functional exercises of lumbar and back muscles and limbs to prevent muscular atrophy and deep vein thrombosis. After 2 weeks, they started wearing waist protection and engaged in partial weight-bearing walking, within 1 month, they started wearing waist or braces. Blood routine, erythrocyte sedimentation rate, and C-reactive protein were rechecked on a monthly basis after discharge.

One month after surgery, a lumbar MRI was reexamined to determine the lesions.

### Clinical and radiologic evaluation

The clinical efficacy was evaluated by visual analog scale (VAS) scores and Japanese Orthopaedic Association (JOA), Oswestry Disability Index (ODI), and standard Macnab's evaluation. The pain intensity was evaluated by VAS (0–10; 0 indicates no pain; 10 indicates severe pain). Improvement based on Macnab's evaluation standard was divided into four following grades: excellent which suggested no pain, no restriction of mobility, possibility to return to normal work, and normal level of activity; good which suggested occasional non-radicular pain, relief of presenting symptoms, possibility to return to modified work; fair which suggested some improvement in functional capacity, still handicapped; poor which suggested continued objective symptoms of root involvement, additional operative invention needed at index level irrespective of the length of postoperative follow-up. Postoperative CT (Toshiba Aquilion, Japan) and MRI (GE HDxt-3T, GE, United States) of vertebrae were examined.

### Statistical Analysis

All statistical analyses were performed by using SPSS 18.0 software (SPPS, Inc.). Data are presented as the mean ± SD. Student *t*-test was used for continuous variables, and Fisher exact test was used to evaluate the differences in clinical outcomes. A *P*-value < 0.05 was considered to be statistically significant.

## Results

A total of 13 patients, 5 male and 8 female, met the inclusion criteria, and their medical records were analyzed. The basic information of patients was shown in Table 1. All patients in this group were followed up for 10–18 months, with an average of 13.69 months. The age of patients ranged from 45 to 69 years, with a median of 58 years. The postoperative low back pain was immediately relieved, and the average hospital stay was (22.00 ± 3.54) days. Three patients, 2 female and 1 male, had different degrees of neurological deficit before surgery, and all recovered to normal at 3 months follow-up after surgery. The VAS score was (6.92 ± 0.64) before surgery, (3.42 ± 0.53) one week after surgery, (2.38 ± 0.58) one month after surgery, and (1.50 ± 0.41) three months after surgery. The VAS scores before and after surgery significantly differed (*P* < 0.05). JOA and ODI indices were significantly lower at 1 week, 1 month, after operation compared to before operation (*P* < 0.05, Table 2). One month after surgery, erythrocyte sedimentation rate and C-reactive protein were all within the normal range (Table 3). According to the MacNAB standard at the last follow-up, 7 patients were excellent, 5 were good, and 1 was poor. No serious complications occurred. The effective postoperative rate was 92.31%. Postoperative MRI showed that the abscesses disappeared (Figures 1G,H).

**Table 2:** The variation in visual analog scale (VAS), Japanese Orthopaedic Association (JOA), and Oswestry Disability Index (ODI) preoperatively and postoperatively (*x* ± *s*, points).

	Preoperative	1 week Postoperative	1 month Postoperative	3 months Postoperative
VAS	6.92 ± 0.64	3.42 ± 0.53*	2.38 ± 0.58*^,^**	1.50 ± 0.41*^,^**
JOA	8.85 ± 1.68	19.31 ± 1.80*	22.31 ± 2.29*^,^**	27.69 ± 2.87*^,^**
ODI	69.23 ± 5.36	46.92 ± 5.36*	25.85 ± 5.86*^,^**	15.77 ± 2.68*^,^**

* Compared with pre-operation *P* < 0.05.

** Compared with one week after surgery, *P* < 0.05.

**Table 3:** Erythrocyte sedimentation rate (ESR), C-reactive protein (CRP), and white blood cell (WBC) count before and after surgery (*x* ± *s*, *n* = 13).

	Preoperative	1 week Postoperative	1 month Postoperative	3 months Postoperative
WBC (×10^9^/L)	12.08 ± 1.69	8.36 ± 1.04*	6.18 ± 0.68*^,^**	5.19 ± 0.72*^,^**
The percentage of neutrophils (%)	83.52 ± 3.45	68.90 ± 3.69*	57.78 ± 3.56*^,^**	49.18 ± 4.76*^,^**
ESR (mm/h)	47.92 ± 4.59	33.00 ± 2.83*	22.38 ± 4.93*^,^**	14.54 ± 3.02*^,^**
CRP (mg/L)	51.85 ± 4.96	18.78 ± 3.72*	6.37 ± 0.79*^,^**	3.62 ± 0.62*^,^**

*Compared with pre-operation *P* < 0.05.

**Compared with one week after surgery, *P* < 0.05.

All patients were irrigated with normal saline containing gentamicin *via* rinsing tube after surgery (2000 ml/24 h). Stop rinsing after 5 days and connect the rinsing tube with a negative pressure suction device. The drainage tube was retained in place until the daily drainage had stopped or reduced to less than 5 ml per day for 3 consecutive days. Drainage tubes were removed 5 days after rinsing in 9 of 13 patients, 7 days in 3 patients, and 10 days after in 1 patient. A case underwent a second percutaneous endoscopic debridement with external drainage for relapse of spinal infection at the treated level during the follow-up period. Finally, the patient experienced significant pain relief without recurrence.

**Figure 2:**
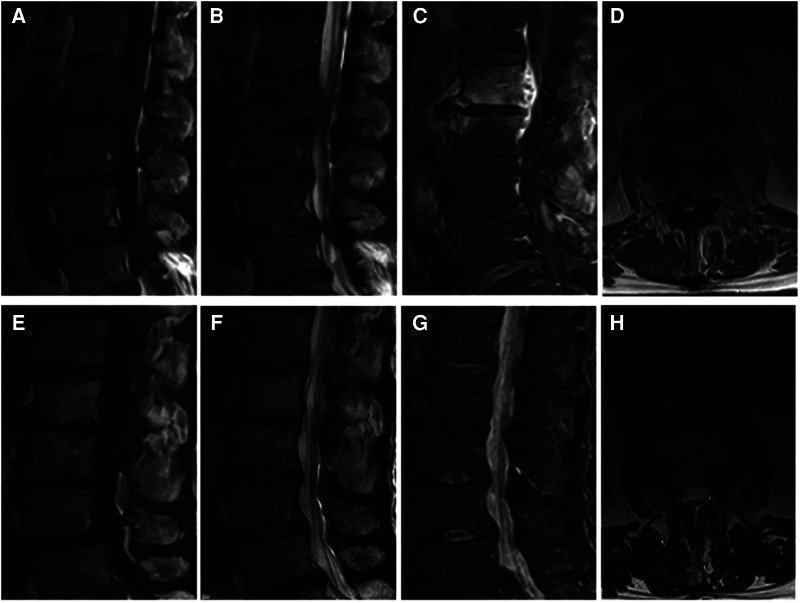
(**A–D**) It presents the MRI scans of patient No. 5. Sagittal and axial magnetic resonance images with L2-L3 intervertebral infection. (**E–H**) It presents the postoperative the MRI scans (9 months after surgery) of patient No. 5. MRI shows that the abscess was disappeared without recurrence. (**A,E**) Sagittal T1WI. (**B,F**) Sagittal T2WI. (**C,G**) Sagittal contrast-enhanced image. (**D,H**) Axial image after surgery.

In this study, blood culture and surgical tissue biopsy were performed on all 13 patients for accurate pathogenetic examination. Among 13 patients, 10 patients had negative blood cultures, and only 3 patients (23.1%) had positive results. The results of bacterial culture in the infected tissues were positive in 7 cases (53.8%), including 4 cases of Escherichia coli, 2 cases of Staphylococcus aureus and 1 case of Mycobacterium tuberculosis. Four patients with Escherichia coli infection were treated with cefoperazone tazobactam for 6 weeks. Two patients with Staphylococcus aureus were treated with cefuroxime for 6 weeks, and continued oral treatment for 6 weeks after discharge. One case of Mycobacterium tuberculosis infection was treated with pasniazide, rifapentine, ethambutol and pyrazinamide for 10 months. The remaining 6 patients with negative bacterial culture results were treated with empiric antibiotics (cefuroxime and levofloxacin). Five of them had a good effect and were treated orally for another 6 weeks after discharge. One patient, who did not respond well to empiric antibiotics, was changed to meropenem and linezolid, and the effect was good. Regular blood routine examination, ESR and CRP of the patients decreased gradually after operation. Typical cases are shown in Figures 1, 2.

## Discussion

Lumbar intervertebral infection is an infection of the lumbar vertebra, intervertebral disc, and soft tissue around the vertebral body caused by pathogenic microorganisms (27). The initial symptoms of a disc infection with an epidural abscess are not specific, and it usually takes 2–6 months from the initial symptoms to the final diagnosis. Delays in diagnosis and treatment are common in all spinal infections because of their delayed early course. Clinical manifestations include intractable back pain, fever, and progressive neurological dysfunction (28). X-ray and CT usually do not show any specific changes in the early stage, while MRI has a high early diagnostic value, which could provide the early basis for clinical diagnosis (3, 11). Traditional treatments of spinal infection mainly include conservative treatment and surgical treatment (29). For lumbar intervertebral infections, conservative treatments such as bed rest, intensive nutrition, and sensitive antibiotics are recommended if there is only a minor disruption or if the infection is at an early stage. Surgical treatment is needed in case antibiotic treatment fails or if there is a progressive spinal deformity, lumbar instability, epidural abscess, or neurological damage (30, 31). Most patients with lumbar intervertebral infection respond well to conservative treatment (32). However, conservative treatment has been associated with long-term bed immobilization, poor patient compliance, bedsores, deep vein thrombosis, pneumonia, and other complications. It may also indirectly cause kyphosis and chronic low back pain (33). Surgical treatment is needed for patients with symptoms of nerve compression, lumbar instability, giant abscess formation, and failure to respond to conservative treatment. The operation can completely remove the lesion, correct the deformity, restore the spinal sequence, improve the neurological function, reduce the recurrence rate, shorten the bedtime, and quickly relieve the pain. Therefore, surgical treatment is currently being more advocated (1, 34).

At present, the traditional open surgical methods mainly include anterior and posterior lesion removal, bone graft fusion and internal fixation, and anterior lesion removal combined with posterior bone graft and internal fixation. For young, healthy patients, open surgery is, in general, effective as it can completely remove the lesion and provide a strong internal fixation. While open surgery can help to achieve interbody fusion and spinal stability, it has also been associated with substantial trauma, thus being more difficult to tolerate by elderly patients with underlying diseases. The use of spinal instruments at the site of infection remains controversial. Although the results of spinal reconstruction surgery with spinal implants are satisfactory, there is still a high incidence of complications, including failure of internal fixation and recurrence of infection (35, 36). Patients with lumbar intervertebral infections often have a variety of basic diseases and low immune function (37). Surgical treatments also include percutaneous suction and flow surgery, CT-guided lesion puncture, and catheter drainage, which can also lead to a relatively satisfactory effect in treating lumbar intervertebral infections (38). Yet, simple catheter lavage and drainage cannot directly remove the lesion tissue, and sometimes the drainage fails because of the sticky texture of the pus.

Percutaneous endoscopic discectomy (PED) is used to treat herniated discs. Over recent years, with the advancement of spinal surgery technology and the development of the minimally invasive concept, intervertebral PED has been widely used in treating lumbar diseases (39). Some studies have also reported that PED can be used to treat spinal infections (25, 40). Using the Kambin safe triangle, the stability of the spinal sequence can be avoided, the lesion tissue can be removed under direct vision, and the drainage tube can be accurately placed in the location of the lesion, thus removing the necrotic tissue and pus through lavage and drainage. In 1997, Haaker et al*.* (41) reported 16 patients with lumbar intervertebral infections who were treated by percutaneous endoscopic disc removal, achieving the satisfactory initial curative effect. Manabu et al. (25) treated 15 patients with spondylitis infection who failed to respond to antibiotic treatment with posterolateral spinal endoscopic debridement, perfusion, irrigation and drainage, and routine antibiotic treatment after surgery. All patients felt obvious pain relief at the end of the operation, and the clinical effect was satisfactory. Moreover, Yang and colleagues (26) performed endoscopic spinal therapy in 32 patients with lumbar intervertebral infections, achieving satisfactory results. In our study, patients were all cleared of infected lesions and necrotic tissues in the intervertebral disc, even the epidural space, under local nerve block anesthesia through foraminal microscopy under direct vision.

**Figure 3:**
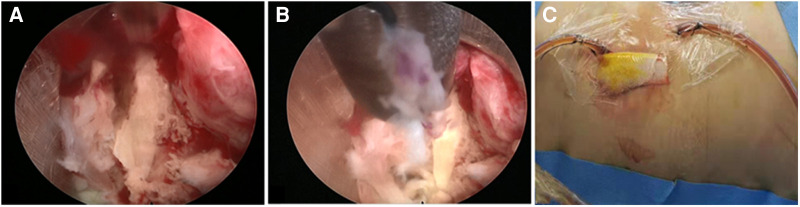
(**A**) Intraoperative photo. (**A**) At the beginning of the PEDI procedure, pus accumulation and granulation tissue at the infected disc level were observed. (**B**) Discectomy forcep, flexible rongeur, and shaver were then inserted through the cannulated sleeve to withdraw as much infected tissue as possible. (**C**) A negative-pressure Hemovac with 2 drainage tubes was inserted through the sheaths for further continuous drainage of the offending pathogens.

Direct endoscopic observation can be used to directly collect enough samples for microbiological examination. Under endoscopic monitoring, infected and dead tissue in the intervertebral disc and even in the epidural space can be eradicated and cleared (Figure 3). During the rinsing process, debris and abscess can be rinsed away. Tissue biopsy of the infected tissue obtained from the operation showed that 7 cases (53.8%) had successfully isolated pathogenic bacteria. Therefore, percutaneous endoscopic lumbar discectomy also has the advantage that the infected and necrotic tissue of the intervertebral disc can be accurately removed under direct endoscopic observation, and sufficient pathological tissue an be obtained, which increases the pathogen detection rate. Postoperative large-caliber negative pressure drainage can continuously remove the pathogen from the infected site. In the current study, the combination of effective debridement with irrigation and whole-course specific antimicrobial therapy led to favorable results.

Because PLD combined with ED is less invasive, easy to perform, and with a low complication rate, we used a nucleus poplar osteotomy forceps to remove the infected disc material and epidural abscess through the posterior approach and then placed two external drainage catheters inside the intervertebral disc. The satisfactory results were consistent with those obtained by surgical decompression. This minimally invasive technique, which is less commonly used than open surgery, can effectively relieve patients' back pain by reducing intra-disc pressure and maintaining adequate stability. These patients can use braces to start physical training as early as possible after surgery. Patients with epidural or paraspinal abscesses may also be treated with this method, avoiding open anterior or posterior decompression. There is often a link between these abscesses and an infected disc, which is the real cause of a spinal infection. Postoperative irrigation, perfusion, and drainage can be used to effectively and continuously remove the pathogens from the infected tissue. The combination is usually more effective than monotherapy. The total course of anti-infection in several studies ranged from 6 to 14.7 weeks, including 3 to 8 weeks of intravenous administration (42). French anti-infection guidelines recommend a minimum treatment period of 6 to 12 weeks (43).

In this study, we used PLD combined with ED to treat disc infection with epidural abscess, achieving satisfactory results consistent with surgical decompression. The postoperative VAS score was significantly reduced, and the pain was significantly relieved. The effective rate was 92.31%, with no serious complications. Compared with surgery, the combination of PLD and ED did not alter the structure or stability of the spine, while it accelerated drainage of infectious materials compared with antibiotics alone. In our study, the lesions were repeatedly irrigated with a large amount of normal saline under an endoscope working sleeve after adequate debridement of the infected disc. Yang et al. (26) reported a group of cases of lumbar spondylitis treated by minimally invasive endoscopic surgery with diluted povidone iodine solution. We used normal saline, which also provided satisfactory back pain relief and infection control without any other complications. In addition, unlike their methods, we used a unilateral working sleeve for endoscopic decompression, clearance, and drainage. After surgery, saline containing gentamicin was given through drainage tube lavage (2000 ml/24 h), which could not only effectively maintain the drug concentration in the lesion, but also fully remove the residual infected tissue and pus, thus improving the anti-infection effect. In addition, compared with the endoscopic bilateral debridement drainage described by Yang (26), unilateral percutaneous endoscopic technique was adequate in our present study. The time required to place unilateral working sleeve is shorter than that of bilateral sleeve, and the damage is less. Postoperative unilateral drainage function may be similar to bilateral drainage.

The present study has some limitations. First, the number of patients treated with PLD in combination with ED was relatively small, which may conceal any advantages or disadvantages of the approach. Second, due to the lack of a control group, the clinical efficacy of PLD combined with ED in treating intervertebral disc infection with epidural abscess cannot be defined as superior to surgery alone. Future studies are needed to address these issues. Third, the follow-up time is short, and the long-term effect remains to be followed up.

Based on our experience, endoscopic surgery effectively relieves the patient's symptoms by reducing the pressure in the disc and maintaining sufficient stability. Direct endoscopy allows sufficient samples to be collected from the infected area for microbiological examination. Under endoscopic monitoring, it can realize the eradication and debridement of intervertebral disc or even epidural space infection and necrotic tissue. Disc debris and cloudy abscesses may be rinsed by the disc sleeve during rinsing. Postoperative negative pressure drainage can continue to suck pathogens out of the infected body. These patients were able to walk with a brace as early as possible after surgery. For elderly patients, especially those with various complications, effective treatment can be achieved under local anesthesia. Indications include single or interstitial epidural abscesses, early spinal infection, and mild or moderate destruction of the vertebral body. However, from the perspective of surgical technique and clinical outcome, the efficacy of this procedure for extensive destruction of the vertebral body and extensive infection may be limited.

## Conclusion

Based on the results of this study, we concluded that PEDI is an effective option for treating lumbar intervertebral infections or combined epidural and paraspinal abscesses. This approach was effective in obtaining a bacteriological diagnosis, alleviating the patient's symptoms, and helping to eradicate infectious spondylitis of the lumbar spine. In these cases, extensive anterior or posterior surgery may not always be necessary, as combining PLD and ED with intravenous antibiotics is an effective and innovative technique for treating intervertebral disc infection complicated with epidural abscess.

## Data Availability

The original contributions presented in the study are included in the article/Supplementary Material, further inquiries can be directed to the corresponding author/s.
